# miR-708 Negatively Regulates TNF*α*/IL-1*β* Signaling by Suppressing NF-*κ*B and Arachidonic Acid Pathways

**DOI:** 10.1155/2021/5595520

**Published:** 2021-03-10

**Authors:** Nicholas J. Monteleone, Carol S. Lutz

**Affiliations:** Department of Microbiology, Biochemistry and Molecular Genetics, Rutgers School of Graduate Studies-RBHS, Newark, NJ 07005, USA

## Abstract

Two pathways commonly dysregulated in autoimmune diseases and cancer are tumor necrosis factor alpha (TNF*α*) and interleukin 1 beta (IL-1*β*) signaling. Researchers have also shown that both signaling cascades positively regulate arachidonic acid (AA) signaling. More specifically, TNF*α*/IL-1*β* promotes expression of the prostaglandin E2- (PGE_2_-) producing enzymes, cyclooxygenase-2 (COX-2) and microsomal prostaglandin E synthase-1 (mPGES-1). Exacerbated TNF*α*, IL-1*β*, and AA signaling have been associated with many diseases. While some TNF*α* therapies have significantly improved patients' lives, there is still an urgent need to develop novel therapeutics that more comprehensively treat inflammatory-related diseases. Recently, researchers have begun to use RNA interference (RNAi) to treat various diseases in the clinic. One type of RNAi is microRNA (miRNA), a class of small noncoding RNA found within cells. One miRNA in particular, miR-708, has been shown to target COX-2 and mPGES-1. Previous studies have also suggested that miR-708 may be a negative regulator of TNF*α*/IL-1*β* signaling. Therefore, we studied the relationship between miR-708, TNF*α*/IL-1*β*, and AA signaling in diseased lung cells. We found that miR-708 negatively regulates TNF*α*/IL-1*β* signaling in nondiseased lung cells, which is lost in diseased lung cells. Transient transfection of miR-708 suppressed TNF*α*/IL-1*β*-induced changes in COX-2, mPGES-1, and PGE_2_ levels. Moreover, miR-708 also suppressed TNF*α*/IL-1*β*-induced IL-6 independent of AA signaling. Mechanistically, we determined that miR-708 suppressed IL-6 signaling by reducing expression of the nuclear factor kappa-light-chain-enhancer of activated B cells (NF-*κ*B) activator inhibitor of nuclear factor kappa-B kinase subunit beta (IKK*β*). Collectively, our data suggest miR-708 regulates TNF*α*/IL-1*β* signaling by inhibiting multiple points of the signaling cascade.

## 1. Introduction

Tumor necrosis factor alpha (TNF*α*) and interleukin 1 beta (IL-1*β*) are proinflammatory cytokines crucial for immune responses [1, 2]. It has been shown that both are necessary for effective host defense from bacterial, viral, and parasitic infections [2, 3]. TNF*α* is produced primarily by macrophages and activated T cells under inflammatory conditions [3]. Through its two cognate receptors, TNF*α* receptor 1/2 (TNFR1/2), TNF*α* regulates proinflammatory responses, cell death signaling, tissue repair, and angiogenesis [3]. Mechanistically, TNF*α* activates nuclear factor kappa-light-chain-enhancer of activated B cells (NF-*κ*B), mitogen-activated protein kinase (MAPK), phosphoinositide 3-kinase (PI3K), Fas-associated protein with death domain (FADD), and TNF receptor-associated factor 1/2 (TRAF1/2) signaling axes [3, 4]. IL-1*β* expression is similarly activated, and its proinflammatory signaling can contribute to IL-R1-mediated NF-*κ*B activation [5]. Given TNF*α* and IL-1*β*'s pivotal role in immune signaling and survival, their dysregulation has been implicated in various diseases.

Loss of TNF*α* signaling control has been shown to contribute to numerous diseases, including rheumatoid arthritis (RA), Crohn's disease, atherosclerosis, cancer, and other autoimmune diseases [3]. Similarly, dysregulated IL-1*β* expression contributes to autoimmune diseases and cancer [5]. There are several approved TNF*α* inhibitors and one IL-1R antagonist used to treat autoimmune diseases, highlighting the importance of this pathway in chronic inflammatory diseases. While these inhibitors have improved patient outcomes, 20–40% of RA patients are nonresponsive to TNF*α* inhibitors and resistance amongst responders is increasing [6]. More recently, researchers have begun to study the therapeutic potential of TNF*α* and IL-1*β* inhibition in cancer. Studies have shown that TNF*α*/IL-1*β* inhibition can suppress protumorigenic immune signaling through reduced angiogenesis, metastasis, and immune evasion [7, 8]. While previous work has highlighted the importance of these cytokines in disease, more efficacious therapies are still needed.

It was recently shown that cyclooxygenase-2 (COX-2), the rate-limiting enzyme in the arachidonic acid signaling pathway, was constitutively overexpressed in ulcerative colitis patients who did not respond to TNF*α* inhibitors [9]. Moreover, COX-2 is also overexpressed in RA and atherosclerosis [10]. Overexpression of COX-2 leads to exacerbated prostaglandin E2 (PGE_2_) production. While PGE_2_ is important for hematopoietic stem cell regeneration, inflammation, and gut integrity, increased levels can promote proliferation, invasion, survival, angiogenesis, and immune evasion in cancer [11, 12]. It has also been well documented that TNF*α* and IL-1*β* signaling promotes COX-2 expression [13–15]. Lastly, there is also significant crosstalk between TNF*α*, IL-1*β*, COX-2, and IL-6, another proinflammatory cytokine implicated in disease [16]. Given these data, it would be worthwhile to examine whether novel COX-2-inhibiting therapies can more efficaciously treat TNF*α*/IL-1*β*-related diseases.

One novel method to inhibit gene expression is through microRNA (miRNA). miRNAs are small (21–25 nts), noncoding RNAs that regulate gene expression posttranscriptionally [17]. miRNAs generally suppress gene expression by targeting the transcript's 3′ UTR with incomplete complimentary, resulting in transcript degradation or translational stalling [17]. One miRNA in particular, miR-708-5p (miR-708), has been shown to target COX-2 in diseased lung cells [18]. miR-708 was also shown to target the NF-*κ*B activator inhibitor of nuclear factor kappa-B kinase subunit beta (IKK*β*), which is intricately involved in canonical TNF*α* signaling [19]. Additionally, forced overexpression of miR-708 in human airway smooth muscle cells decreased expression of asthma-related genes [20]. miR-708 also decreased alcohol-induced liver inflammation by targeting zinc finger E-box-binding homeobox 1 (ZEB-1) upregulation of TNF*α* and IL-6 [21]. Lastly, injections of miR-708 mimic into a rodent RA model ameliorated the RA index by inhibiting WNT signaling [22]. Given the various immunoregulatory functions of TNF*α*, IL-1*β*, IL-6, and COX-2, paired with the anti-inflammatory functions of miR-708, we decided to investigate whether miR-708 negatively regulates TNF*α* and IL-1*β* signaling in disease.

In this article, we show that miR-708 expression is temporally induced by TNF*α*/IL-1*β* in nondiseased lung cells. Conversely, miR-708 expression is nonresponsive to TNF*α*/IL-1*β* in diseased lung cells. Exogenous miR-708 in nonresponsive lung cells reduced TNF*α*/IL-1*β*-induced changes in COX-2, mPGES-1, PGE_2_, and IL-6 levels. Lastly, we determined that miR-708 is inhibiting TNF*α*/IL-1*β* signaling dually by suppression of AA and NF-*κ*B signaling in diseased lung cells. Collectively, these data suggest miR-708 is a potent negative regulator of TNF*α*/IL-1*β*, which is lost in diseased lung cells.

## 2. Methods

### 2.1. Cytokine Treatment for Lung Cells

First, 4 × 10^5^ (A549/Beas2b) cells were plated in 60 mm dishes. The next morning, 3 mL of serum-free DMEM (4 mM L-glutamine and 1% Penicillin/Streptomycin) containing recombinant human 50 ng/mL TNF*α* (PeproTech, Rocky Hill, NJ) and 10 ng/mL IL-1*β* (PeproTech) was added to cells for 0–48 hours, followed by RNA/protein isolation or media removal for ELISA. If treated with miR-708, BAY-11-7082, or CEL, cells were pretreated O/N before TNF*α*/IL-1*β* treatment.

### 2.2. Enzyme-Linked Immunosorbent Assay (ELISA)

A549 PGE_2_ levels in cell culture media were analyzed using the PGE_2_ Express ELISA Kit (500141, Cayman Chemical, Ann Arbor, MI) per manufacturer's instructions. Media was removed, and cells were incubated for 20 min with serum-free media containing 10 *μ*M arachidonic acid (Cayman Chemical) in serum-free DMEM. Collected media was centrifuged at 5000 g × 10 min, 4°C. Media was transferred to new tubes and then centrifuged at 2000 g × 10 min, 4°C, before being transferred to new tubes. Before analysis, samples were diluted 10x with 1x ELISA buffer. Absorbance was read using the SpectraMax M2 plate reader (Molecular Devices, San Jose, CA). PGE_2_ levels were measured in technical duplicates, normalized to total protein levels, and are an average of ≥3 biological replicates. When probing for IL-6, we used a similar kit from Cayman Chemical (501030) in accordance with the manufacturer's protocol. In contrast to the PGE_2_ ELISA, complete media was collected from cells at each time point indicated. Centrifugation was performed as stated above. Media was not diluted prior to performing the IL-6 ELISA. IL-6 levels were measured in technical duplicates, normalized to total protein levels, and are an average of ≥3 biological replicates.

### 2.3. Mammalian Cell Culture

A549 cells (ATCC, Manassas, VA) were grown in Dulbeccoʼs Modified Eagle's Medium (DMEM, MilliporeSigma) supplemented with 10% FBS, 4 mM L-glutamine, and 1% Penicillin/Streptomycin. All cells were incubated at 37°C in a 5% CO_2_ incubator and subcultured using 0.05% Trypsin, 0.53 mM EDTA (Corning, Corning, NY).

### 2.4. miRNA, BAY-11-7082, and CEL Treatments

A549 cells were seeded in 6-well plates at 3 × 10^5^ cells per well. Synthetic versions of hsa-miR-708-5p and nontargeting miRNAs were purchased from Horizon Discovery, Waterbeach, United Kingdom; hsa-miR-708-5p mature miRNA sequence: 5′-AAGGAGCUUACAAUCUAGCUGGG-3′, accession #: MIMAT004926. Horizon Discovery's miRIDIAN microRNA Mimic Negative Control #1 (sequence is not provided) was used as a nontargeting miRNA. This miRNA has a scrambled sequence with no predicted targets in the human transcriptome. Twenty-four hours after seeding, cells were transiently transfected with synthetic miRNAs at 25 nM (unless stated otherwise) using INTERFERin (Polyplus, Berkeley, CA) according to the manufacturer's protocol. Using the same seeding protocol, cells were treated with 10 *μ*M BAY-11-7082 (Abcam) or 10 *μ*M celecoxib (MilliporeSigma) in complete medium [23]. Fresh BAY-11-7082 was added after 24 hours. Cells were treated for a total of 48 hours prior to RNA/protein isolation or media removal for ELISA.

### 2.5. NF-*κ*B Promoter Assay

We purchased the NanoLuc Reporter Vector with NF-*κ*B Response Element (http://pNL3.2.NF-*κ*B-RE, Promega, Fitchburg, WI). Briefly, this reporter construct has an NF-*κ*B RE upstream of the *NLucP* luciferase gene. First, we plated 1 × 10^5^ A549 cells into each well of a 12-well plate. 24 hours later, we treated cells with mock, 25 nM miR-708, or 10 *μ*M BAY-11-7082 (explained in miRNA, BAY-11-7082, and CEL Treatments). 24 hours later, cell media was replaced with serum-free DMEM. The following morning, cells were treated with TNF*α*/IL-1*β* (+/- BAY-11-7082) for 5 hours. Cells were then analyzed using the Nano-Glo Luciferase Assay System (Promega) per manufacturer's protocol. Data were background subtracted from each treatment (TNF*α*/IL-1*β* alone, +miR-708, +BAY-11-7082) at each time point (0, 5 h). Samples were then normalized to total protein and TNF*α*/IL-1*β* 0 h treatment (noninduced control). Luciferase data represent the average of ≥3 biological replicates.

### 2.6. Quantitative Real-Time PCR (qRT-PCR)

Complementary DNA (cDNA) was synthesized by reverse transcription of RNA using the miScript II RT Kit (Qiagen, Venlo, Netherlands). miRNA-specific cDNA was created using HiSpec buffer, while mRNA-specific cDNA was created using HiFlex buffer. qRT-PCR was performed using a Bio-Rad CFX96 Real-Time C1000 Touch Thermal Cycler. miRNA cycling conditions were as follows: (1) 95°C for 15 min, (2) 40 cycles of 94°C for 15 s, 55°C for 30 s, and 70°C for 30 s (collection step). mRNA cycling conditions were similar, except for adjusted annealing temperatures on a primer-by-primer basis. miR-708-5p, U6 snRNA, and COX-2 primers were purchased from Qiagen, while ODZ4, mPGES-1, and GAPDH primers were purchased from OriGene. Amplification was performed using the miScript SYBR Green PCR Kit (Qiagen). No template and no reverse transcriptase controls, as well as melt curve analysis, were implemented to ensure samples/primers were not contaminated. Quantitative comparative C_T_ (*ΔΔ*C_T_) analysis was used to analyze gene expression changes relative to U6 snRNA (miRNA) or GAPDH (mRNA). qRT-PCR data represent the average of ≥3 biological replicates. Each sample was measured with *n* ≥ 2 technical replicates per target gene per independent experiment.

### 2.7. RNA Isolation

Total RNA was isolated from cells using TRIzol (Invitrogen, Carlsbad, CA) following the manufacturer's protocol. Samples were further purified with the Direct-zol RNA Miniprep Kit (Zymo Research). RNA was quantified using the SimpliNano Spectrophotometer (GE, Boston, MA).

### 2.8. Statistical Analysis

We used Prism 7 software to perform one-way ANOVA and Student's *t*-test to determine significant differences. Where indicated, the nonparametric tests were used to determine statistical significance. *p* values less than 0.05 were considered significant.

### 2.9. Western Blot Analysis

Media was removed from treated cells and lysed in RIPA buffer (50 mM Tris at pH 8.0, 150 mM NaCl, 1% Nonidet P-40, 0.5% sodium deoxycholate, 0.1% SDS, and 0.1% protease inhibitor). The cells/supernatant were scraped off wells, collected, and then centrifuged at 14000 × g for 15 min at 4°C. Protein concentration was determined using the DC Protein Assay (Bio-Rad, Hercules, CA). 25 *μ*g of protein was loaded onto 10% SDS-PAGE gels, separated by electrophoresis, and transferred onto PVDF membrane (VWR) for 2 hours at 4°C. Blots were blocked with 5% nonfat milk+PBSt (5% nonfat dry milk, 1x PBS, 0.1% Tween-20 (MilliporeSigma)) for 1 hour at room temperature (RT). Primary antibody incubations against human COX-2 (160112, Cayman Chemical, Ann Arbor, MI), IKK*β* (ab264239, Abcam), mPGES-1 (ab180589, Abcam), and GAPDH (HRP-60004, Proteintech, Rosemont, IL) were performed overnight at 4°C per manufacturer's recommended dilutions. Blots were washed with PBSt 3x for 5 minutes each and then exposed to secondary HRP conjugated secondary antibodies (goat anti-mouse H+L (31430, Thermo Fisher, Waltham, MA); goat anti-rabbit H+L (31460, Thermo Fisher)) for 1 hour at RT. Blots were developed using Clarity Western ECL Substrate (Bio-Rad) on the ChemiDoc MP Imaging System (Bio-Rad). Western blot images are representative of ≥3 biological replicates.

## 3. Results

### 3.1. miR-708 Expression Is Induced by TNF*α*/IL-1*β* in Nondiseased Lung Cells

Proinflammatory cytokines have extensive functions in regulating chronic inflammatory-related diseases. Two important proinflammatory cytokines dysregulated in disease are TNF*α* and IL-1*β*. Several articles have determined that miR-708 represses NF-*κ*B, TNF, and arachidonic acid (AA) signaling. Given these data, we investigated whether miR-708 was a posttranscriptional regulator of these cytokines.

**Figure 1:**
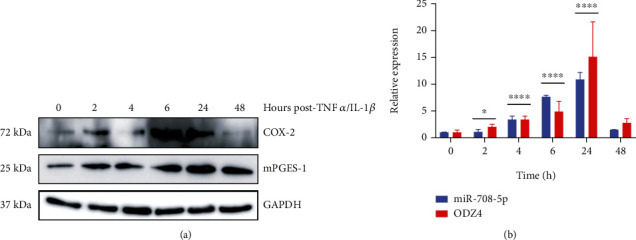
TNF*α*/IL-1*β* modulates COX-2, mPGES-1, and miR-708 expression temporally in nondiseased lung cells. (a) Representative western blot of COX-2 and mPGES-1 protein expression from 0 to 48 hours after TNF*α*/IL-1*β* exposure in Beas2b cells. GAPDH served as a loading control. (b) RT-qPCR of mature miR-708 (blue) and ODZ4 (red) mRNA expression in Beas2b cells 0–48 hours post-TNF*α*/IL-1*β* exposure. miR-708 expression was normalized to U6 snRNA while ODZ4 mRNA was normalized to GAPDH mRNA and analyzed using the 2^-*ΔΔ*CT^ method. miR-708 and ODZ4 levels were compared to 0 h treatments when determining significance; ^∗^*p* < 0.05 and ^∗∗∗∗^*p* < 0.0001, *n* ≥ 3.

First, COX-2 and mPGES-1 protein expression is induced by TNF*α*/IL-1*β* signaling in Beas2b (nondiseased) lung cells (Figure 1(a)). Beas2b cells are immortalized, nontransformed lung epithelial cells. As previously reported, TNF*α*/IL-1*β* induces COX-2 in a time-dependent manner, usually returning to baseline 48–72 hours posttreatment [24]. Confirming previous studies, Beas2b COX-2 and mPGES-1 protein expression is strongly induced 6 hours post-TNF*α*/IL-1*β* treatment and returns to baseline by 48 hours (Figure 1(a)). Interestingly, mature miR-708 expression in Beas2b cells is highly responsive to TNF*α*/IL-1*β* treatment, peaking by 24 hours and coming back to baseline by 48 hours (Figure 1(b), *p* < 0.05, *n* = 3). We also examined how miR-708 levels were increased. To do this, we measured *Odz4* expression. miR-708 is located within intron 1 of the *ODZ4* gene, and researchers have shown that miR-708 expression is controlled through the *ODZ4* promoter [25]. In Beas2b cells, *Odz4* mRNA expression mirrors miR-708 expression, suggesting TNF*α*/IL-1*β* regulates miR-708 transcriptionally through the *ODZ4* promoter (Figure 1(b), *p* < 0.05, *n* = 3).

### 3.2. miR-708 Expression Is Nonresponsive to TNF*α*/IL-1*β* in Diseased Lung Cells

**Figure 2:**
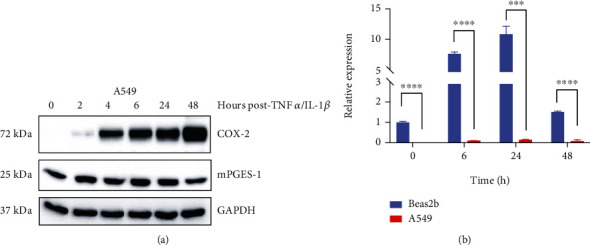
miR-708 and AA signaling regulation is uncoupled in TNF*α*/IL-1*β*-modulated diseased lung cells. (a) Representative western blot of COX-2 and mPGES-1 protein expression from 0 to 48 hours after TNF*α*/IL-1*β* exposure in A549 cells. GAPDH served as a loading control. (b) RT-qPCR of mature miR-708 in Beas2b (blue, nondiseased) and A549 (red, diseased) cells 0–48 hours post-TNF*α*/IL-1*β* exposure. miR-708 expression was normalized to U6 snRNA and analyzed using the 2^-*ΔΔ*CT^ method. ^∗∗∗^*p* < 0.001 and ^∗∗∗∗^*p* < 0.0001, *n* = 3.

We repeated our experiments in A549 (diseased) lung cells, which are a lung adenocarcinoma cell line. While cancerous, researchers have used this cell line to study other inflammatory-related diseases. A549 cells treated with TNF*α*/IL-1*β* dramatically increased COX-2 protein levels through 48 hours, with no resolution observed (Figure 2(a)). Conversely, we saw nonsignificant TNF*α*/IL-1*β*-induced increases in miR-708 expression in A549 cells (Figure 2(b)*p* = n.s., *n* = 3). When paired with Beas2b miR-708 expression changes, we see that A549 miR-708 levels are significantly lower at baseline (Figure 2(b), *p* < 0.0001, *n* = 3). We have previously shown that reduced miR-708 expression in A549 cells is due to *ODZ4* promoter methylation [18]. Moreover, A549 miR-708 levels never increased above 15% of baseline Beas2b expression after TNF*α*/IL-1*β* treatment, and miR-708 levels are significantly lower at every time point in A549 cells (Figure 2(b), *p* < 0.001, *n* = 3). These data suggest that *ODZ4* promoter methylation prevents TNF*α*/IL-1*β*-induced miR-708 expression, disconnecting the negative feedback loop in diseased lung cells and leading to unresolved COX-2 signaling. As a control, we performed viability (WST-1) analysis on Beas2b and A549 cells untreated or TNF*α*/IL-1*β* treated and found that TNF*α*/IL-1*β* treatment did not affect cellular viability in either cell line (Supplemental Figure 1). Given these data, we next investigated if transient transfection of exogenous miR-708 could suppress TNF*α*/IL-1*β*-induced changes in A549 cells.

### 3.3. miR-708 Reduces TNF*α*/IL-1*β*-Induced Changes in Diseased Lung Cells by Suppressing NF-*κ*B Signaling

**Figure 3:**
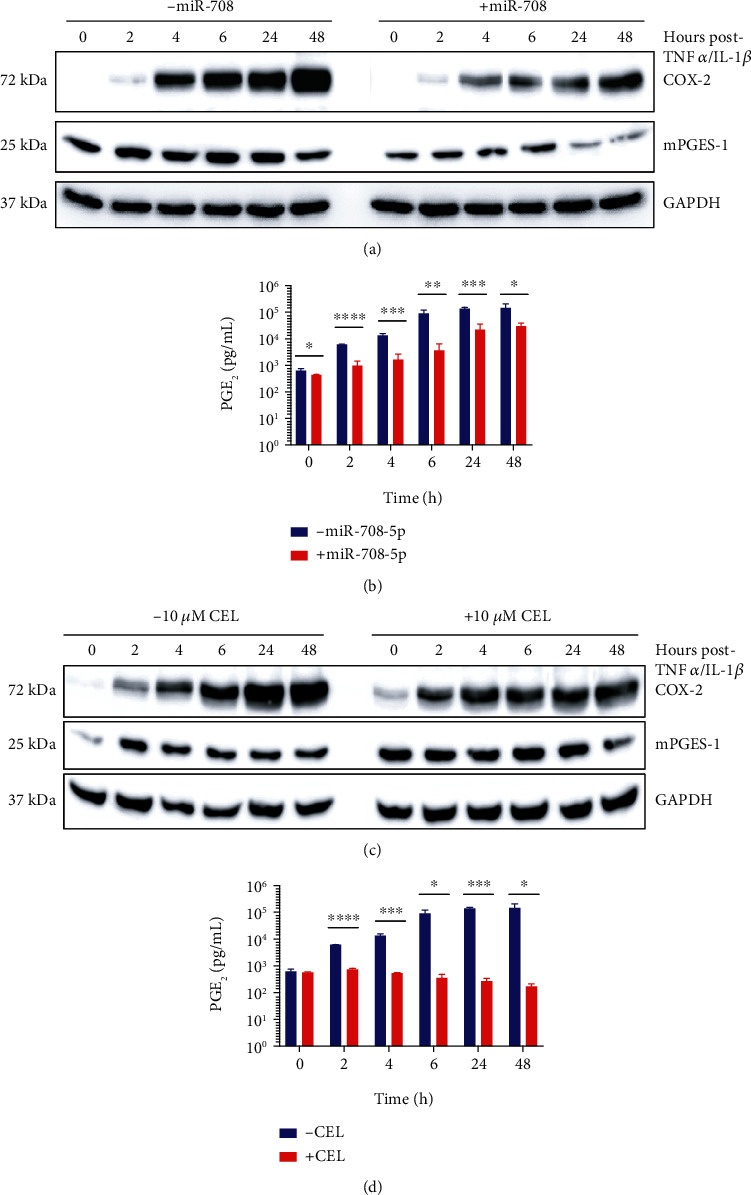
miR-708 suppresses TNF*α*/IL-1*β*-induced arachidonic acid signaling partially through a positive feedback loop in diseased lung cells. (a) Representative western blot examining COX-2 and mPGES-1 protein expression in A549 cells transiently transfected with mock (left) or 25 nM miR-708 (right) in combination with TNF*α*/IL-1*β* for 0–48 hours. (b) ELISA measuring exogenous PGE_2_ levels in A549 cells transiently transfected with mock (blue) or 25 nM miR-708 (red) and treated with TNF*α*/IL-1*β* for 0–48 hours. (c) Representative western blot examining COX-2 and mPGES-1 protein expression in A549 cells cotreated with vehicle (left) or 10 *μ*M CEL (right) and TNF*α*/IL-1*β* for 0–48 hours. (d) ELISA measuring exogenous PGE_2_ levels in A549 cells cotreated with vehicle (blue) or 10 *μ*M CEL (red) and TNF*α*/IL-1*β* for 0–48 hours. ^∗^*p* < 0.05, ^∗∗^*p* < 0.01, ^∗∗∗^*p* < 0.001, and ^∗∗∗∗^*p* < 0.0001; *n* ≥ 3.

We transiently transfected A549 cells with mock (-miR-708) or synthetic miR-708 and then exposed cells to TNF*α*/IL-1*β* for 0–48 hours. As seen in Figure 3(a), A549 cells without miR-708 treatment had robust induction of COX-2 protein expression in a time-dependent manner. A549 cells treated with synthetic miR-708 had a significant reduction in both COX-2 and mPGES-1 protein levels across all TNF*α*/IL-1*β* time points (Figure 3(a)). We also measured PGE_2_ release by ELISA and observed significantly decreased PGE_2_ levels at every time point in miR-708-treated A549 cells compared to mock samples (Figure 3(b), *p* < 0.05, *n* ≥ 3). PGE_2_ has been shown to form a positive feedback loop to enhance COX-2 expression and other cytokines.

**Figure 4:**
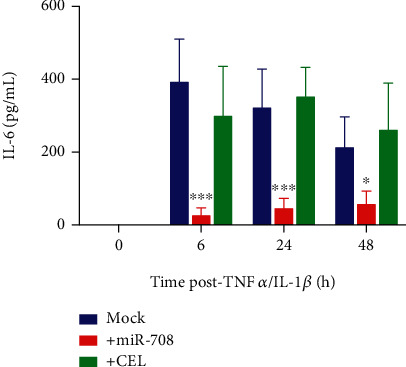
miR-708 suppresses TNF*α*/IL-1*β* induced IL-6 expression independent of COX-2 signaling. ELISA measuring exogenous IL-6 levels in A549 cells cotreated with vehicle (blue), 25 nM miR-708 (red), or 10 *μ*M CEL (green) and TNF*α*/IL-1*β* for 0–48 hours. ^∗^*p* < 0.05 and ^∗∗∗^*p* < 0.001, *n* ≥ 3.

We inhibited COX-2 production of PGE_2_ with celecoxib (CEL) and repeated our experiments. While CEL efficiently suppressed PGE_2_ production (Figure 3(d), *p* < 0.05, *n* ≥ 3), it only partially reduced TNF*α*/IL-1*β* regulation of COX-2 protein expression (Figure 3(c)). Next, we measured IL-6 production, as researchers have shown that it is directly regulated by COX-2/mPGES-1-derived PGE_2_. While miR-708 prevented TNF*α*/IL-1*β*-induced IL-6 expression, CEL had no effect on IL-6 levels after TNF*α*/IL-1*β* treatments (Figure 4, *p* < 0.05, *p* = n.s., *n* ≥ 3). These data reveal that miR-708 is suppressing IL-6 production independent of AA signaling. As previously discussed, TNF*α*/IL-1*β* has been shown to induce IL-6 both through AA signaling and independent of COX-2, mPGES-1, or PGE_2_. Given these data, we next examined the mechanism by which miR-708 is suppressing IL-6 levels in diseased lung cells.

**Figure 5:**
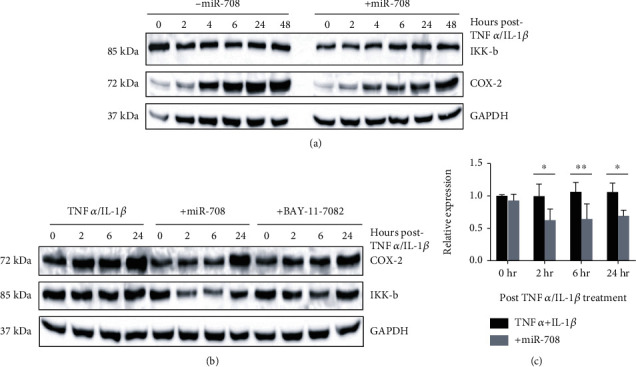
miR-708 and NF-*κ*B inhibition decreases IKK*β* and COX-2 protein expression, respectively, in TNF*α*/IL-1*β*-treated diseased lung cells. (a) Representative western blot examining COX-2 and IKK*β* (IKK-b) protein expression in A549 cells transiently transfected with mock (left) or 25 nM miR-708 (right) in combination with TNF*α*/IL-1*β* for 0–48 hours. (b) Representative western blot examining COX-2 and IKK*β* (IKK-b) protein expression in A549 cells transiently transfected with mock (left), 25 nM miR-708 (middle), or 10 *μ*M BAY-11-7082 in combination with TNF*α*/IL-1*β* for 0–24 hours. (c) Quantification of IKK*β* protein expression from representative western blot (b) and others (data not shown). IKK*β* expression was normalized to GAPDH protein expression and TNF*α*/IL-1*β* 0 h (left black bar). ^∗^*p* < 0.05 and ^∗∗^*p* < 0.01, *n* ≥ 3.

Studies have shown that IL-6 can be regulated through the NF-*κ*B pathway [26]. Moreover, miR-708 was also shown to target the NF-*κ*B activator IKK*β*, leading to decreased NF-*κ*B-regulated gene expression [19]. Therefore, we examined whether miR-708-mediated IKK*β* suppression could be responsible for reduced IL-6 levels in miR-708-treated A549 cells. First, we measured IKK*β* protein expression in TNF*α*/IL-1*β*-treated A549 cells with mock (-miR-708) or miR-708 (+miR-708). We found that miR-708 treatment decreased A549 IKK*β* protein levels up to 24 hours after TNF*α*/IL-1*β* treatment (Figures 5(a) and 5(c), *p* < 0.05, *n* = 3). Next, we compared A549 cells treated with TNF*α*/IL-1*β*+mock, miR-708, or the IKK*β* inhibitor BAY-11-7082 and repeated our western blot. BAY-11-7082-treated cells decreased TNF*α*/IL-1*β*-mediated COX-2 expression in a similar manner to miR-708 (Figure 5(b)). While it is important to show miR-708 reduces IKK*β* protein expression, we must also show miR-708 decreases NF-*κ*B activity.

**Figure 6:**
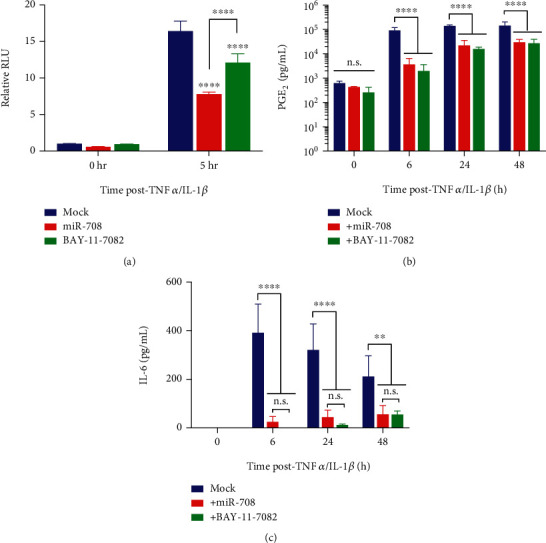
miR-708 suppresses PGE_2_ and IL-6 secretion by inhibiting NF-*κ*B signaling in diseased lung cells. (a) Relative NF-*κ*B promoter reporter NanoLuc luciferase activity in A549 cells. Relative luciferase activities were measured in response to mock (blue), 25 nM miR-708 (red), or 10 *μ*M BAY-11-7082 (green) treatment for 0 or 5 h. Data were normalized to total protein and mock 0 h samples. Differences were compared between samples within 0 and 5 h time points. (b) ELISA measuring exogenous PGE_2_ levels in A549 cells cotreated with vehicle (blue), 25 nM miR-708 (red), or 10 *μ*M BAY-11-7082 (green) and TNF*α*/IL-1*β* for 0–48 hours. (c) ELISA measuring exogenous IL-6 levels in A549 cells cotreated with vehicle (blue), 25 nM miR-708 (red), or 10 *μ*M BAY-11-7082 (green) and TNF*α*/IL-1*β* for 0–48 hours. ^∗∗^*p* < 0.01 and ^∗∗∗∗^*p* < 0.0001, *n* ≥ 3.

**Figure 7:**
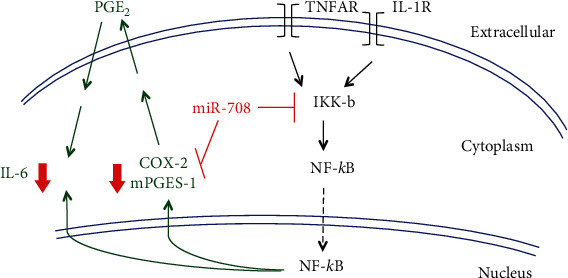
Proposed model of miR-708-mediated negative regulation of TNF*α*/IL-1*β* signaling in diseased lung cells. Model representing the repressive effects of miR-708 on TNF*α*/IL-1*β*-mediated regulation of arachidonic acid and IL-6 signaling in A549 cells. Black arrows represent activated signaling pathways, while black dotted lines represent miR-708-inhibited signaling cascades. Green lettering and arrows indicate AA signaling, and red lines represent miR-708 target suppression.

To achieve this, we utilized the pNL3.2.NF-*κ*B-RE reporter luciferase assay. Briefly, this reporter construct has an NF-*κ*B response element (RE) upstream of the *NLucP* luciferase gene. Once NF-*κ*B is shuttled to the nucleus, it binds to the NF-*κ*B RE, activating NLucP expression. Changes in NLucP expression can then be quantified via luminescence assays. We transfected A549 cells with mock, 25 nM miR-708, or 10 *μ*M BAY-11-7082, followed by the pNL3.2.NF-*κ*B-RE construct the following day. The next morning, we treated these cells with TNF*α*/IL-1*β* for 0 or 5 h and then measured luminescence. We found that TNF*α*/IL-1*β*+mock A549 cells had a 16x increase in luminescence after 5 hours (Figure 6(a)). While BAY-11-7082 significantly reduced TNF*α*/IL-1*β*-mediated NF-*κ*B activation and luminescence by 28%, miR-708 had a greater suppressive effect than BAY-11-7082 (Figure 6(a), *p* < 0.0001, *n* = 3). These data suggest miR-708 is indeed suppressing TNF*α*/IL-1*β*-mediated NF-*κ*B activation and downstream signaling. Given these data, we repeated the PGE_2_ and IL-6 ELISAs in A549 cells. We found that BAY-11-7082 significantly reduced A549 PGE_2_ production after TNF*α*/IL-1*β* treatment similarly to miR-708 (Figure 6(b), *p* < 0.0001, *n* = 3). Lastly, BAY-11-7082 also suppressed IL-6 production in A549 cells post-TNF*α*/IL-1*β* treatment in a time-dependent manner (Figure 6(c), *p* < 0.01, *n* = 3). There was not a significant difference in IL-6 expression between miR-708 and BAY-11-7082 treatments at every time point, suggesting that miR-708's effects can be attributed to NF-*κ*B signaling inhibition. Collectively, these data reveal that miR-708 is suppressing IL-6 production independent of AA signaling in lung cancer cells. IL-6 is not a predicted miR-708 target, suggesting miR-708's regulation of IL-6 is indirect. In Figure 7, we propose a model for how miR-708 is suppressing TNF*α*/IL-1*β* signaling in A549 cells based on our data and published studies. We surmise that miR-708 suppresses TNF*α*/IL-1*β* through multiple mechanisms, by inhibiting both NF-*κ*B and AA signaling in diseased lung cells.

## 4. Discussion

TNF*α* and IL-1*β* are proinflammatory cytokines crucial for immune responses, especially important for host defense from bacterial, viral, and parasitic infections [1, 3]. Uncontrolled TNF*α*/IL-1*β* signaling has been shown to contribute to numerous diseases, including RA, Crohn's disease, atherosclerosis, cancer, and autoimmune diseases [3]. While there are several approved TNF*α* inhibitors used to treat autoimmune diseases, a significant subset of patients is nonresponsive to TNF*α* inhibitors or becomes resistant to treatment [6]. Interestingly, it was found that COX-2 is overexpressed in RA and ulcerative colitis patients and is constitutively overexpressed in TNF*α* inhibitor nonresponders [9, 10]. IL-6 is also overexpressed in many tumors and has been shown to regulate various aspects of oncogenesis [27, 28]. Furthermore, researchers have shown that miR-708 dampens TNF*α* signaling, possibly by inhibiting NF-*κ*B signaling [29, 30]. Therefore, we examined the ability of miR-708 to mitigate downstream TNF*α*/IL-1*β*-related signaling in disease.

In this study, we first confirmed a previous work that showed TNF*α* upregulated COX-2 and mPGES-1 protein expression within 6 hours and returned to baseline by 48 hours in normal lung cells (Figure 1(a)). Interestingly, miR-708 was temporally upregulated similar to other TNF*α*-dependent genes in nondiseased lung cells (Figure 1(b)). In diseased lung cells, TNF*α*/IL-1*β* highly upregulated COX-2 expression, but AA signaling was never restored to baseline (Figure 2(a)). Conversely, TNF*α*/IL-1*β* had no effect on miR-708 expression in lung cancer cells (Figure 2(b)). This suggests that a novel miR-708 negative feedback loop may be lost in diseased lung cells. Therefore, we tested the ability of exogenous miR-708 to suppress TNF*α*/IL-1*β* signaling in diseased lung cells.

We found that miR-708 significantly reduced TNF*α*/IL-1*β*-induced COX-2, mPGES-1, and PGE_2_ levels in A549 cells (Figures 3(a) and 3(b)). Reduced AA signaling can partially be contributed to inhibition of an AA signaling positive feedback loop (Figures 3(c) and 3(d)). Next, we tested whether miR-708 could inhibit downstream COX-2-dependent genes, specifically IL-6. We found that miR-708-treated diseased lung cells had significantly lower IL-6 secretion post-TNF*α*/IL-1*β* exposure compared to TNF*α*/IL-1*β* treatment alone (Figure 4). However, our data suggest that miR-708 suppression of IL-6 expression is independent of AA signaling in diseased lung cells (Figure 4). Given these data, we examined whether miR-708 was modulating the IL-6 regulator NF-*κ*B.

We found that miR-708 decreased IKK*β* protein expression, a potent NF-*κ*B activator (Figure 5). Using a NF-*κ*B promoter luciferase construct, we determined that miR-708 significantly reduced TNF*α*/IL-1*β*-induced NF-*κ*B transcriptional activity (Figure 6(a)). Lastly, we inhibited NF-*κ*B signaling using BAY-11-7082 and found that NF-*κ*B inhibition reduced PGE_2_ and IL-6 production similarly to miR-708 treatment (Figures 6(b) and 6(c)). This finding supports the hypothesis that miR-708's regulation of IL-6 is through the NF-*κ*B pathway, not AA signaling. Given these data, we created a model showing miR-708's dampening effects on TNF*α*/IL-1*β* in lung cells (Figure 7). We found that miR-708 acts dually to suppress TNF*α*/IL-1*β* signaling by inhibiting both AA and NF-*κ*B signaling. Moreover, loss of miR-708 in diseased lung cancer may contribute to uncoupled TNF*α*/IL-1*β* signaling. Lastly, these data suggest miR-708 may act as a novel therapeutic to treat TNF*α*/IL-1*β*-related diseases. These data provide the foundation for further exploring the role of miR-708 in TNF*α*/IL-1*β*-related diseases.

While we have laid the foundation for expanding research on the precise functions of miR-708 in TNF*α*/IL-1*β* signaling, several questions remain. First, how is TNF*α* inducing miR-708 expression in normal lung cells? Previous researchers have shown that encourages the expression/activity of the miR-708 regulators CHOP, CTBP2, C/EBP-*β*, and CTCF [31–34]. Given these data, there are multiple avenues by which TNF*α*/IL-1*β* may stimulate miR-708 expression. Given that TNF*α* suppresses GR*α*, MYC, and E2F1 signaling, known miR-708 regulators, it is unlikely that these transcription factors are involved in our study. On the other hand, CHOP and C/EBP-*β* stimulate miR-708 expression and are TNF*α*-responsive transcription factors. Therefore, CHOP and C/EBP-*β* are possibly transcriptional regulators of TNF*α*/IL-1*β*-induced miR-708 expression. While this provides a plausible signaling axis, we have not identified why miR-708 is not upregulated after TNF*α*/IL-1*β* treatment in diseased lung cells.

There are several possible explanations for how TNF*α*/IL-1*β*-mediated activation of miR-708 is lost in diseased lung cells. First, transcriptional activators may not be expressed in these cells. Second, the *ODZ4* promoter may be inaccessible to transcription factors due to promoter methylation and chromatin remodeling. Third, miR-708-negative regulators may be preventing transcriptional activation. Previous studies have shown that CHOP signaling is inhibited or defective in cancer [35]. Additionally, C/EBP-*β* is necessary for effective lung cell inflammatory responses and is lost in cancer [36, 37]. Therefore, loss of CHOP and C/EBP-*β* activity may prevent activation of miR-708 expression in diseased lung cells. Secondly, it has been previously shown that the *ODZ4* promoter, which miR-708 is under the control of, is hypermethylated in diseased lung cells [18]. This may be through EZH2, which is positively influenced by TNF*α*/IL-1*β* signaling and has been shown to hypermethylate the *ODZ4* promoter in cancer, resulting in repressed miR-708 expression [38, 39]. Lastly, two negative regulators of miR-708 that are positively regulated by TNF*α*/IL-1*β*, CTBP2 and CTCF, are overexpressed in lung immune-related diseases [40, 41]. Collectively, these studies suggest that the loss of miR-708 responsiveness to TNF*α*/IL-1*β* stimulation may be a multifaceted mechanism. Loss of transcriptional activators, overexpression of negative regulators, and *ODZ4* promoter hypermethylation may collectively inhibit miR-708 expression in diseased lung cells. The consequences of this miR-708 suppressive network may be profound. TNF*α*-induced chronic inflammatory states frequently result in epithelial cell transformation. Loss of TNF*α*-negative regulators such as miR-708 further propagates TNF*α* signaling, increasing the risk of transformation and autoimmune disease. Therefore, miR-708 may be a crucial TNF*α*/IL-1*β* signaling-suppressing miRNA involved in early stage inflammatory-related oncogenesis and autoimmunity. Given these proposed circumstances, it would be justifiable to study the therapeutic potential of miR-708 in inflammatory-related cancers and autoimmune diseases.

Given the data we have uncovered, as well as TNF*α*/IL-1*β*'s therapeutic relevance in autoimmune diseases and cancer, it would be beneficial to examine miR-708's ability to reduce autoimmune-related inflammation and oncogenesis. TNF*α*, IL-1*β*, and IL-6 are all prominent genes implicated in immune evasion. The ability of miR-708 to suppress TNF*α*, IL-1*β*, IL-6, and AA signaling axes may provide a more comprehensive therapeutic intervention compared to current targeted therapies. Additionally, significant subsets of patients receiving TNF*α* inhibitors become resistant to treatment. In these populations, researchers showed that COX-2 was overexpressed [9]. Moreover, COX-2 inhibition overcame TNF*α* resistance in these ulcerative colitis patients [9]. While COX-2 inhibitors have shown promise in treating autoimmune diseases and cancer, they have unacceptable long-term side effect profiles. Therefore, using miR-708 to treat these populations may provide a fresh therapeutic alternative. While the efficacy and safety of miR-708 is unknown, future studies will uncover the therapeutic potential of miR-708 in treating TNF*α*/IL-1*β*-related diseases.

## 5. Conclusions

Our data suggests miR-708 is a potent negative regulator of TNF*α*/IL-1*β* signaling in lung epithelial cells. Loss of endogenous miR-708 expression may contribute to unchecked TNF*α*/IL-1*β* signaling, leading to inflammatory-related diseases. Reestablishing miR-708 levels may counteract TNF*α*/IL-1*β*-induced inflammation, ultimately resolving disease phenotype.

## Data Availability

To request data, please contact Dr. Carol S. Lutz at http://lutzcs@njms.rutgers.edu.

## Supplementary Materials

Supplementary Materials Supplementary Figure 1 TNF*α*/IL-1*β* treatment does not alter viability in lung cells. Nondiseased (Beas2b, (A)) and diseased (A549, (B)) lung cells were untreated (blue) or treated with TNF*α*/IL-1*β* (red) for 48 hours. Metabolic rates were measured using the WST-1 assay. Samples were analyzed at 0, 2, 4, 6, 24, and 48 hours and normalized to total protein; *n* ≥ 3.
