# Supplementary material for: miR-708 Negatively Regulates TNFα/IL-1β Signaling by Suppressing NF-κB and Arachidonic Acid Pathways
Source: Mediators Inflamm. 2021 Mar 10;2021:5595520. doi: 10.1155/2021/5595520 (PMC7969122; doi:10.1155/2021/5595520)
Supplement: Supplementary Materials — Supplementary Figure 1 TNFα/IL-1β treatment does not alter viability in lung cells. Nondiseased (Beas2b, (A)) and diseased (A549, (B)) lung cells were untreated (blue) or treated with TNFα/IL-1β (red) for 48 hours. Metabolic rates were measured using the WST-1 assay. Samples were analyzed at 0, 2, 4, 6, 24, and 48 hours and normalized to total protein; n ≥ 3. [file 5595520.f1.docx]

**Supplementary Figure 1. TNFα/IL-1β treatment does not alter viability in lung cells.** Non-diseased (Beas2b, [**A**]) and diseased (A549, [**B**]) lung cells were untreated (blue) or treated with TNFα/IL-1β (red) for 48 hours. Metabolic rates were measured using the WST-1 assay. Samples were analyzed at 0, 2, 4, 6, 24, and 48 hours and normalized to total protein. n ≥ 3.
